# Salt-Induced Damage is Alleviated by Short-Term Pre-Cold Treatment in Bermudagrass (*Cynodon dactylon*)

**DOI:** 10.3390/plants8090347

**Published:** 2019-09-13

**Authors:** Jibiao Fan, Jilei Xu, Weihong Zhang, Maurice Amee, Dalin Liu, Liang Chen

**Affiliations:** 1 College of Animal Science and Technology, Yangzhou University, Yangzhou, Jiangsu 225009, China; 006298@yzu.edu.cn (J.F.); xjl333753@163.com (J.X.); zwh2019wbgcas@163.com (W.Z.); 2 Key Laboratory of Plant Germplasm Enhancement and Specialty Agriculture, Wuhan Botanical Garden, Chinese Academy of Sciences, Wuhan 430074, China; maurice.amee@gmail.com; 3 University of Chinese Academy of Sciences, 19 Yuquan Road, Beijing 100049, China

**Keywords:** salt stress, cold priming, bermudagrass, fluorescence transient, gene expression

## Abstract

Excess salinity is a major environmental stress that limits growth and development of plants. Improving salt stress tolerance of plants is important in order to enhance land utilization and crop yield. Cold priming has been reported to trigger the protective processes in plants that increase their stress tolerance. Bermudagrass (*Cynodon dactylon*) is one of the most widely used turfgrass species around the world. However, the effect of cold priming on salt tolerance of bermudagrass is largely unknown. In the present study, wild bermudagrass was pre-treated with 4 °C for 6 h before 150 mM NaCl treatment for one week. The results showed that the cell membrane stability, ion homeostasis and photosynthesis process which are usually negatively affected by salt stress in bermudagrass were alleviated by short-term pre-cold treatment. Additionally, the gene expression profile also corresponded to the change of physiological indexes in bermudagrass. The results suggest that cold priming plays a positive role in improving salt stress tolerance of bermudagrass.

## 1. Introduction

Excess salinity is one of the main environmental stresses limiting growth and development, productivity and distribution of plants throughout the world. Salt stress causes changes in a series of cellular processes, photosynthesis process and gene expression in plants [1,2,3]. For instance, salt stress inhibits plant growth and damages the cell membrane [4,5]. Previous studies have revealed that electrolyte leakage (EL) increased dramatically in perennial ryegrass (*Lolium perenne*) [6]. The Na^+^ was accumulated, while K^+^ content decreased in Arabidopsis [7]. The change of ion accumulation will affect the metabolic process further [8]. Simultaneously, the chlorophyll content decreased in plant under salt stress [9]. In response, the plants have evolved several mechanisms such as: cell membrane system, antioxidant system and photosynthetic system, in order to protect themselves against salt stress [9,10,11]. Photosynthesis is one of the most essential physiological processes in plants that are sensitive to salt stress [12]. When a leaf is exposed to dark conditions, distinguishable changes of chlorophyll a fluorescence intensity can be detected, referred to the Kautsky effect [13]. Information about the different redox states of PSII and efficiencies of electron transfer in the photosynthetic electron transfer chain is contained in Chl a fluorescence transient [14]. For this reason, chlorophyll fluorescence analysis is widely used in plant physiology studies [15]. Generally, when Chl *a* fluorescence transient is plotted on a logarithmic time scale, four steps known as O-J-I-P could be clearly observed [13]. The four steps reflect different reactions in the photosynthetic pathway. Generally, the reaction centers (RCs) are totally open at O step, so the highest photochemistry yield and minimal Chl a fluorescence yield are measured. At the O-J step, the primary quinone electron acceptor Q_A_ was reduced to Q_A_^−^; the J-I-P steps reflect the reduction of PQ pool; and the RCs are totally closed at P step, so the maximal fluorescence yield is measured. It was reported that the energy flux and electron transport were hindered in *Brassica juncea* by salt stress [16].

When plants are exposed to stress conditions, physiological and molecular changes occur in plant cells to counter the stresses. For example, the compatible solutes are accumulated as osmolytes, the membrane compositions are altered for enhanced fluidity, and the protective proteins are synthesized [17]. Cold acclimation is a natural physiological process in plants when treated with low but above freezing temperatures, enabling the plant to survive in freezing winter [18]. During this process, the cell wall is modified and the proteomic and metabolomic profilings of the plant change [19,20]. The freezing tolerance of alfalfa (*Medicago sativa*), eucalypt (*Eucalyptus nitens*) and tomato (*Solanum lycopersicum*) were improved after cold acclimation treatment [21,22,23]. It has been proven that CBF genes are involved in cold acclimation in Arabidopsis, and that the CBFs are also important for salt tolerance of plant [24]. Therefore, single short cold stress can improve cold tolerance of plants, and this process is called cold priming [25].

Advanced technologies such as high-throughput phenotyping and sequencing are contributed to identifying many stress response related genes in plant [26]. Transcriptomic analysis has showed that numerous genes are related to stress priming in plant [27]. For example, the genes of some crucial enzymes such as *NADP-ME*, *SLP* and *Peroxidase* were upregulated in peach after pre-cold treatment [28]. *ECA4* is one of the Ca^2+^ ATPase genes, it is reported that the Ca^2+^ ATPase genes were remarkably upregulated by cold and salt stress in plant [29]. RAN is a small GTPase that plays important roles in nuclear transporting and assembling, it can affect plant response to cold and salt stress, and *RAN1* gene is reported to be involved in cold resistance of rice (*Oryza sativa*) [30]. Moreover, the MHX gene which is a Mg^2+^/H^+^ exchanger gene [31] is regulated by cold and salt stress in plant according to our previous study of transcriptomic analysis [32]. In addition to the ion homeostasis-related genes, photosynthesis-related genes are also regulated by stress conditions in plants. The chloroplast encoded genes expression such as *psbA*, *psbB* and *psbE* are regulated by salt stress in plant [33]. Bermudagrass (*Cynodon dactylon*) is a warm-season turfgrass species. It is widely used in golf courses, gardens, sports fields and home lawns. However, the global change induced many environmental stresses, and it is very important for grass engineering to improve the stress tolerance [34]. The stress tolerance mechanisms of bermudagrass are largely unknown. Therefore, investigating the change in bermudagrass under stress conditions is necessary to understanding its stress tolerance mechanism. According to our previous study, numerous stress response genes were up-regulated after short-term cold acclimation [35]. Some of these genes are not only related to cold but also to other stress factors such as salt stress [36]. Hence, in the present study, the bermudagrass was treated with salt stress after pretreatment with low temperature to investigate whether short-term cold acclimation affects its salt stress tolerance.

## 2. Results

### 2.1. The Effect of Short-Term Pre-Cold Treatment on Cell Membrane Stability in Bermudagrass under Salt Stress

**Figure 1:**
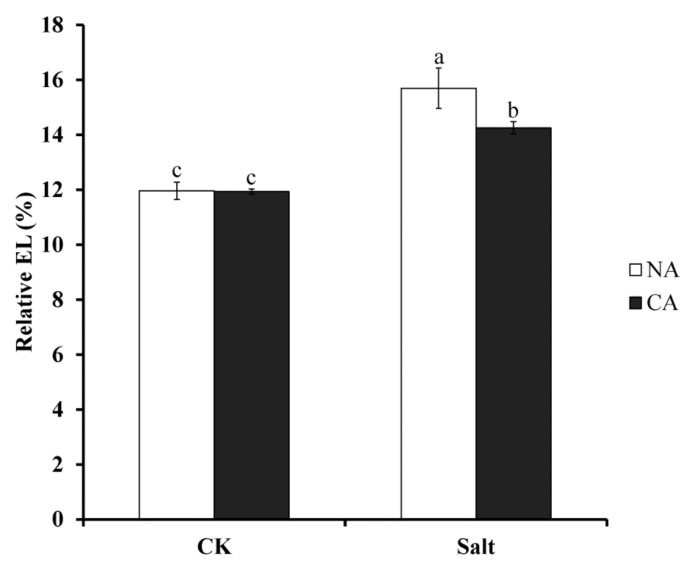
Electrolyte leakage (EL) in bermudagrass under different treatments. Mean values and standard deviation (SD) were calculated from three independent experiments. Different letters indicated significant difference (*p* < 0.05) based on one-way analysis of variance (ANOVA), Duncan’s multiple range test. Bars show SD. CK = control; NA = non-cold treatment; CA = pre-cold treatment for 6 h.

EL increase is usually induced by loss of membrane stability; to investigate the effect of cell membrane stability of pre-cold treatment under salt stress, relative EL was measured. The results showed that the relative EL under salt stress was 31.2% and 19.2% higher than that of the control in non-cold treatment and pre-cold treatment plants, respectively (Figure 1). There was no significant difference between non-cold treatment and pre-cold treatment plants under normal conditions. This suggests that short-term pre-cold treatment had a positive effect on bermudagrass tolerance to salt stress.

### 2.2. The Effect of Short-Term Pre-Cold Treatment on Ion Homeostasis in Bermudagrass under Salt Stress

**Figure 2:**
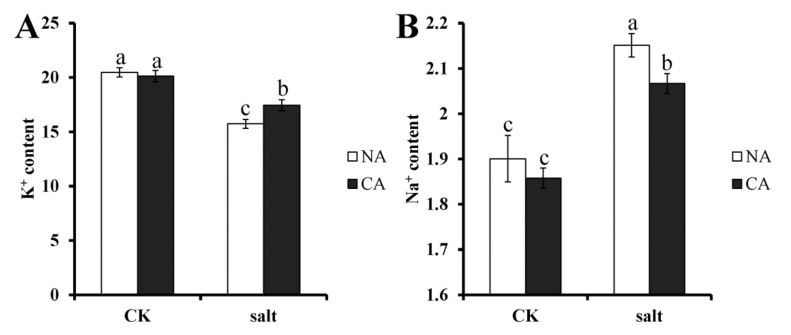
Ion content in bermudagrass under different treatments. (**A**). K^+^ content; (**B**). Na^+^ content. Mean values and standard deviation (SD) were calculated from three independent experiments. Different letters indicated significant difference (*p* < 0.05) based on one-way analysis of variance (ANOVA), Duncan’s multiple range test. Bars show SD. CK = control; NA = non-cold treatment; CA = pre-cold treatment for 6 h.

Ion homeostasis in plants is usually affected by salt stress. To investigate whether pre-cold treatment has an effect on ion homeostasis in bermudagrass under salt stress, K^+^ and Na^+^ concentrations were measured. The results showed that K^+^ content was remarkably decreased in the leaves of bermudagrass, 23.1% lower than that of control, when exposed to salt stress. However, after pre-cold treatment under salt stress, K^+^ content was 13.3% lower than that of control in bermudagrass (Figure 2A). Contrary to the result of K^+^ content, the concentration of Na^+^ increased significantly in bermudagrass leaf, 13.2% higher than that of control, under salt stress. After pretreatment with short-term low temperature, the Na^+^ content decreased to 11.2% higher than that of control under salt stress (Figure 2B). There was no significant difference between non-cold treatment and pre-cold treatment plants under control condition.

### 2.3. The Effect of Short-Term Pre-Cold Treatment on Chlorophyll a/b (Chl a/b) in Bermudagrass under Salt Stress

**Figure 3:**
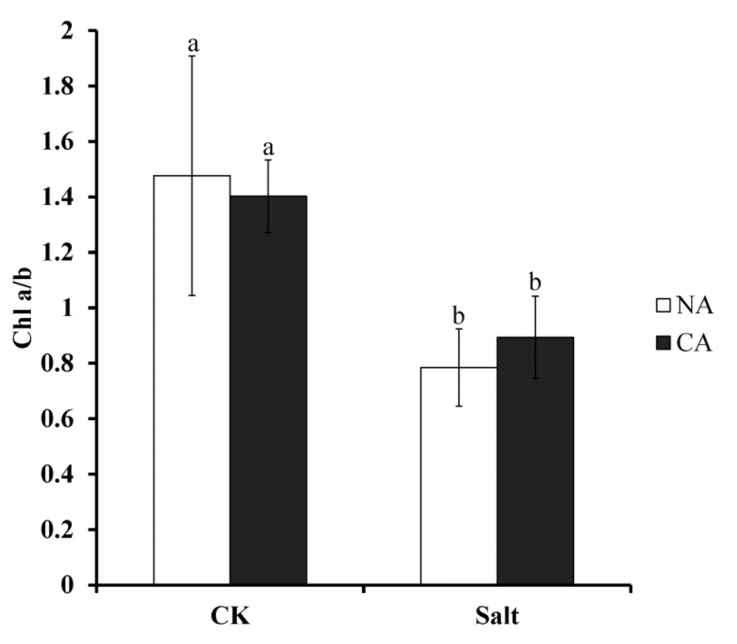
Chlorophyll (Chl) a/b ratio of bermudagrass under different treatments. Mean values and standard deviation (SD) were calculated from three independent experiments. Different letters indicate significant difference (*p* < 0.05) based on one-way analysis of variance (ANOVA), Duncan’s multiple range test. Bars show SD. CK = control; NA = non-cold treatment; CA = pre-cold treatment for 6 h.

As the pigment for photosynthesis, chlorophyll is very sensitive to stress conditions. To investigate the change of chlorophyll in bermudagrass after short-term pre-cold treatment under salt stress, Chl a/b ratio was measured. The results showed that this ratio was severely depressed to 46.9% lower than that of control when bermudagrass was exposed to salt stress. However, the ratio increased in the plants when pretreated by short-term low temperature, still under salt condition. Generally, although there was no statistical difference, the Chl a/b ratio was 14% higher in pre-cold treated plants compared with non-cold treated plants when exposed to salt stress (Figure 3).

### 2.4. The Effect of Short-Term Pre-Cold Treatment on Chl a Fluorescence Transient (OJIP) in Bermudagrass under Salt Stress

**Figure 4:**
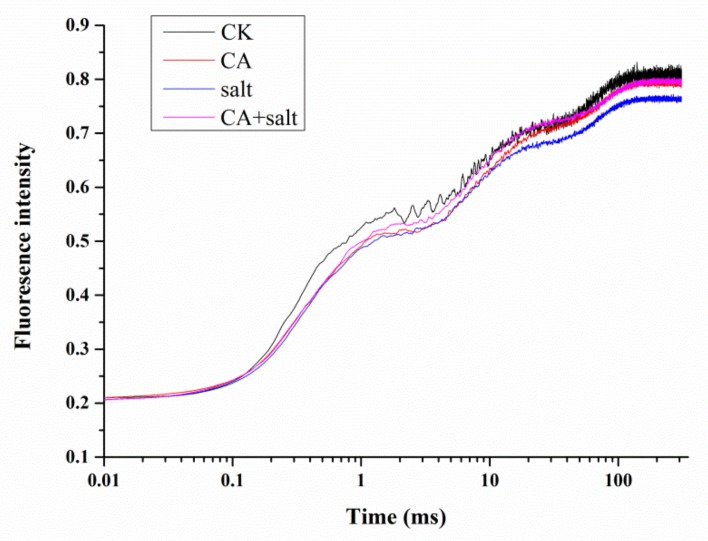
Chlorophyll (Chl) *a* fluorescence transient in bermudagrass under different treatments. CK = control; CA = pre-cold treatment for 6 h; salt = 150 mM NaCl treatment for 1 week.

To investigate the activity of photosystem II (PSII) in bermudagrass after short-term pre-cold treatment under salt stress, Chl *a* fluorescence transient was measured and JIP test was analyzed. The results showed that the OJIP curve declined remarkably as compared to control when the plants were exposed to salt stress. However, when pretreated with low temperature under salt stress, the OJIP curve was significantly higher than in the plants not pretreated with low temperature (Figure 4 and Figure S1). This result revealed that short-term pre-cold treatment could change the Chl *a* fluorescence transient in bermudagrass under salt stress.

**Figure 5:**
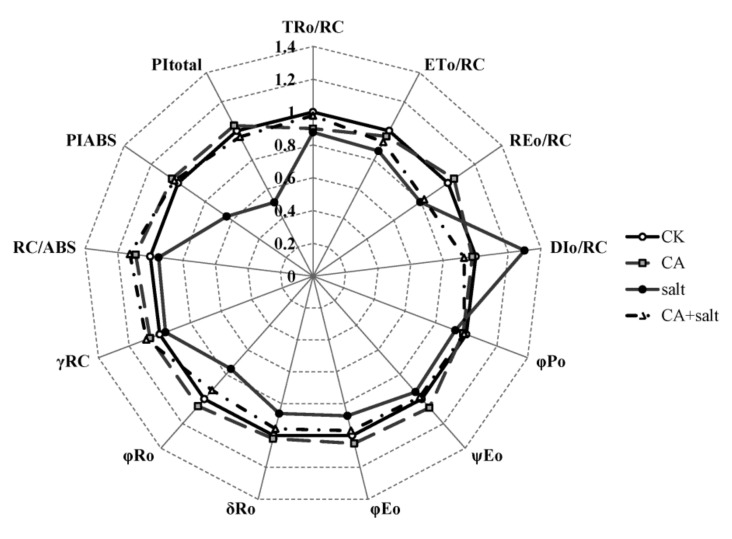
Parameters of JIP test analysis in bermudagrass under different treatments. Mean values were calculated from three independent experiments. CK = control; NA = non-cold treatment; CA = pre-cold treatment for 6 h.

To further understand the change of PSII activity in bermudagrass after salt stress treatment, the JIP test was conducted. The result is shown in Figure 5 and Table S1. Parameters of specific energy fluxes; TR_O_/RC, ET_O_/RC and RE_O_/RC decreased dramatically in PSII of bermudagrass when exposed to salt stress compared to control. TR_O_/RC was also decreased after pre-cold treatment. However, under salt stress conditions, TR_O_/RC and ET_O_/RC increased in the plants that were pretreated with cold. Contrary to the change of other parameters, DI_O_/RC increased significantly in bermudagrass under salt stress, while it dramatically declined in the plants pretreated with cold. This result implied that short-term pre-cold treatment could affect the energy fluxes in PSII of bermudagrass under salt stress. For the parameters of quantum yields and efficiencies, it was shown that φPo, ψEo, φEo, δRo, γRC and RC/ABS were found to be decreased in the plants that were treated with salt stress compared to control. These parameters increased in short-term pre-cold and salt-treated plants compared to only salt-treated plants. This result revealed that short-term pre-cold treatment had an effect on not only energy fluxes, but also quantum yields and efficiencies in PSII of bermudagrass under salt stress. The performance indexes (PI), including PI_ABS_ and PI_toal_, were important parameters, because they were composite indicators to reveal the responses of photosynthetic apparatus. The result showed that both of these two parameters decreased significantly in bermudagrass when exposed to salt stress. However, the performance index in plants that were treated with short-term pre-cold, increased remarkably compared to the plant treated with salt stress alone.

### 2.5. Gene Expression

**Figure 6:**
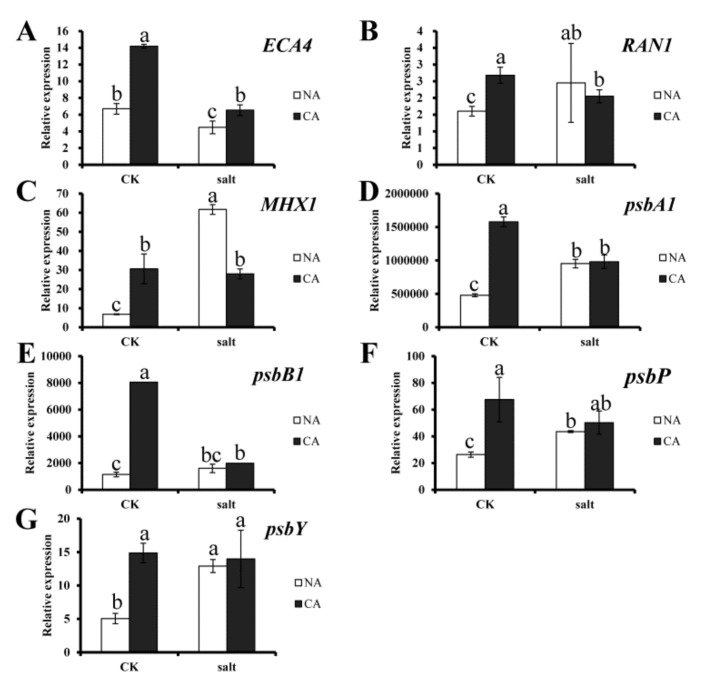
Expression of candidate genes in bermudagrass under different treatments. (**A**) *ECA4* gene expression; (**B**) *RAN1* gene expression; (**C**) *MHX1* gene expression; (**D**) *psbA1* gene expression; (**E**) *psbB1* gene expression; (**F**) *psbP* gene expression; (**G**) *psbY* gene expression. Quantitative real-time PCR was repeated three times. One-way analysis of variance (ANOVA), Duncan’s multiple range test was used to determine statistical differences. Bars show SD.

To investigate the gene expression profile in bermudagrass under salt stress, expression of 7 candidate genes, including *ECA4*, *RAN1*, *MHX1*, *psbA1*, *psbB1*, *psbP* and *psbY*, were measured. The results showed that all of the genes were upregulated significantly in bermudagrass under different treatment compare to that in control. Generally, all of the genes were dramatically upregulated by short-term pre-cold treatment. However, *ECA4* was downregulated by salt stress, and it was recovered after pre-cold treatment. For other genes, comparing between pre-cold treatment and salt stress, the ion transporting related genes such as *RAN1* and *MHX1* were more sensitive to salt stress, while the photosynthesis-related genes, including *psbA1*, *psbB1*, *psbP* and *psbY*, were more sensitive to pre-cold treatment (Figure 6). These results suggest that the genes expressions were regulated by pre-cold and salt treatment.

## 3. Discussion

Abiotic stresses such as salt, heat and drought can cause severe damages in plants. Salt stress can inhibit plant growth and decrease crop yield. When exposed to salt stress, as sessile organisms, plants have to respond and adapt to the adverse conditions. Investigating the mechanism of salt stress response and finding a way to improve the stress tolerance of plants is crucial. Recently, several studies have reported that not only chemical agents but also pre-acclimation can enhance abiotic stress tolerance of plant [37,38]. It was reported that low level salinity pre-treatment could increase salt tolerance of *Zea mays* [38], high blue light pre-treatment could improve UV stress tolerance of pepper (*Capsicum annuum*) [39], and cold acclimation pre-treatment could enhance cold tolerance of bermudagrass (*Cynodon dactylon*) [35]. However, the effect of cold priming on other stress responses in plants is largely unknown. The present study demonstrates that short-term pre-cold treatment could increase the salt tolerance of bermudagrass.

When exposed to salt stress, growth and development of plants is inhibited, and the metabolic processes in plant cells change [40]. Plenty of reactive oxygen species (ROS) accumulate in plant cells, which induces peroxidation of lipids, proteins and nucleic acids [41,42]. Hence, the cell membrane stability could be damaged leading to cell destruction [43]. The present study reveals that the relative EL of bermudagrass dramatically increases under salt stress. This implies that the cell membrane of bermudagrass is damaged by the adverse condition. Additionally, the growth rate of the plants under salt stress was significantly lower than that of control (data not shown). This result corresponds to that of sesame (*Sesamum indicum*) [44] and tomato (*Lycopersicon esculentum*) [5]. Many studies have shown that the activities of antioxidant enzymes would increase to scavenge the ROS induced by salt stress in plants [45,46,47].

As opposed to other stresses, salt stress can cause osmotic stress and ion toxicity in plants. The water loss and growth inhibition takes place first, a phenomenon termed ion-independent response. With continued salt stress, Na^+^ accumulates to toxic concentrations and premature senescence occurs then, referred to as ion-dependent response [48,49]. Ion homeostasis is easily disturbed by salt stress. Na^+^ and Cl^−^ ions have been shown to significantly accumulate in cells of Arabidopsis and maize (*Zea mays*), while the concentration of K^+^ and Ca^2+^ decreased, which induced the Na^+^/K^+^ ratio increase [50,51]. The accumulation of Na^+^ and Cl^-^ causes toxic effects on plant cell and induces cell death in the plant. The present study also shows that the Na^+^ content increased while K^+^ content decreased in bermudagrass under salt stress. This implies that ion homeostasis in cells of bermudagrass was affected by salt stress, which would have negative effects on the plant’s growth and nutrient element absorption [52].

Photosynthesis plays a crucial role in plants and it is sensitive to stress conditions [53]. As the infrastructure of photosynthesis, chlorophyll directly determines the photosynthetic efficiency of plants. Salt stress causes a decrease of chlorophyll synthesis and increase of chlorophyllase-mediated degradation [54]. It was reported that the accumulation of ROS in plant cells causes damage of Chl *a*, which induces a decrease in Chl a/b ratio in *Hydrilla verticillata* [55]. Consistent with this, the present study demonstrates that Chl a/b ratio decreased in bermudagrass after salt stress treatment. The damage to Chl *a* would result in malfunction of photosystem (PS), especially the PSII. The Chl *a* fluorescence transient and JIP test are usually applied to estimate change of PSII of plants under stress conditions [56]. The results in this study showed that the OJIP transient of bermudagrass was significantly changed by salt stress. This is in agreement with the results reported in *Lemna gibba* [57]. In addition, according to JIP test analysis, several parameters of energy flux and electron transport in PSII of bermudagrass were significantly changed. TR_O_/RC, ET_O_/RC and RE_O_/RC reflect the flux of trapped energy, electron transport and the reduction of end electron acceptors at PSI acceptor side per RC, respectively [14]. The decrease of these parameters suggested that the RC activity was depressed by salt stress. The energy was dissipated as fluorescence which was reflected by increase of DI_O_/RC. In addition, the parameters of quantum yields and efficiencies were also changed in bermudagrass under salt stress. For instance, φPo, the maximum quantum yield of PSII, is usually used to evaluate the plant performance under stress conditions [58]. φPo dramatically declined in *Lemna gibba* under salt stress [57]. The present study also agrees with this result. The other parameters also detected a decrease in bermudagrass when it was exposed to salt stress. The parameters include: ψEo, which is the probability that the electrons move further than Q_A_^-^; φEo, which represents the quantum yield for electron transport; δRo, which represents the probability of an electron transferred from carriers to acceptors at PSI side; γRC, which represents the probability of a Chl molecule functioning as RC; and RC/ABS, representing the probability of RCs reduced by Q_A_ per antenna Chl. This result is also consistent with a previous study in *Ageratina adenophora* [59]. The performance indexes, including PI_ABS_ and PI_total_, are very sensitive to environmental stresses and are widely used to assess the photosynthetic functions of plant [60]. The performance indexes of bermudagrass were significantly decreased under salt stress, as shown in this study. A similar result was also reported in Arabidopsis under osmotic stress [61]. Above all, the results suggested that the salt stress negatively affected the electron transfer and energy transduction in PSII of bermudagrass.

When plants are exposed to a specific stress the tolerance to another stress could be improved, a phenomenon termed cross tolerance [62,63]. Previous studies have reported that short-term pre-cold treatment is involved in modifying the response to future stresses in plants, a process known as cold priming [64]. In plants, the cold-shock responses share the majority of pathways with heat-shock responses [65]. Many physiological traits such as antioxidant systems and osmolyte content are modulated [66]. The results of this study show that not only cell membrane stability, but also ion homeostasis and photosystem in bermudagrass, are all improved by cold priming under salt stress. In addition, gene expression is regulated by cold priming under stress condition [66]. Previous study showed that cold response related genes could be upregulated in bermudagrass when it was treated with cold stress [67]. The present study also shows that the candidate genes were remarkably upregulated by short-term pre-cold treatment. Among the genes, *ECA4* is a Ca^2+^ transporter gene, *RAN1* is a Cu^2+^ transporter gene, *MHX1* is a sodium/calcium exchanger gene, and *psbA1*, *psbB1*, *psbP* and *psbY* are photosynthesis related genes in bermudagrass. Up-regulation of these genes implies that the ion transporting processes are triggered by cold priming and salt stress in bermudagrass. The photosynthesis related genes were significantly upregulated by short-term pre-cold treatment. This suggests that cold priming maybe related to alleviating the salt stress damage in bermudagrass through regulating the photosynthesis process.

## 4. Conclusions

Salt stress is an important environmental stress that negatively affects physiological processes in bermudagrass. The salt stress tolerance is enhanced by synthetic compounds [68]. The results of this study showed the salt-induced damage in bermudagrass can be alleviated by short-term pre-cold treatment.

It was shown that the relative EL was significantly decreased after short-term pre-cold treatment under salt stress. It is reasonable that the relative EL will increase under stress conditions because of the injury of cell membrane. The decrease of relative EL suggested the improvement of cell membrane stability after pre-cold treatment.

The results of ion homeostasis in bermudagrass revealed that Na^+^ concentration decreased under salt stress after cold priming treatment. The K^+^ concentration increased accordingly. This implied that the ion homeostasis in bermudagrass is improved by cold priming when the plants are exposed to salt stress.

The salt stress response of photosystem in bermudagrass is improved by cold priming. Under salt stress, the ratio of chlorophyll *a* and *b* slightly increased after short-term cold treatment. Additionally, Chl *a* fluorescence (OJIP) transient and JIP test analysis showed that the parameters of energy flux and electron transport in PSII of bermudagrass was improved by cold priming under salt stress. This implied that the salt tolerance of photosynthesis process in bermudagrass was enhanced by cold priming.

Furthermore, the expression of ion homeostasis and photosystem related genes were upregulated by cold priming. This result corresponded to the change of the physiological indexes.

## 5. Materials and Methods

### 5.1. Plant Materials and Growth Conditions

The bermudagrass (*Cynodon dactylon*) used in the present study was collected from wild field of Guangxi Province in China. It was sensitive to salt stress as demonstrated by the pre-experiment. To cultivate the seedlings, 30 stolons were planted in each pot (10 cm tall and 8 cm in diameter). All the pots were filled with brown coal soil and sand (1:1). The pots were kept in a greenhouse with temperature of 31/23 °C (day/night), the photoperiod was 12 h, the photosynthetically active radiation (PAR) on the canopy was 240 μmol·m^−2^·s^−1^ on average. The plants were cultivated in the pots for three weeks, which allowed the grass to be established. During the period, the plants were fertilized adequately with full-strength Hoagland nutrient solution every day.

### 5.2. Treatments

There are four different treatments—only salt, only cold, cold and salt treatment, and non-cold and salt treatment—in this study. Three repeats were set in each treatment. The established bermudagrass was pretreated with 4 °C for 6 h in a growth chamber (HP300GS-C; Wuhan Ruihua Instrument and Equipment Co., Wuhan, China). The PAR was 240 μmol·m^−2^·s^−1^, and the relative humidity was 60%. To avoid the thermal effect of the light, cold light illuminators were applied in this study. After cold acclimation, the bermudagrass was transferred to 25 °C, 14 h/10 h (day/night) photoperiod conditions and treated with 150 mM NaCl for 1 week. For salt treatment, 500 mL 150 mM NaCl solution was irrigated into the soil at the beginning of treatment. To avoid the solution draining out, a tray was placed under each pot. During the treatment, fully extended leaves after 12 h treatment were collected for RNA extraction. Other samples were collected after 7 days of salt stress treatment.

### 5.3. Measurements

#### 5.3.1. Electrolyte Leakage

Electrolyte leakage (EL) was measured following Hu et al. (2012) [69]: 0.1 g of fully extended leaves was sampled and washed with deionized water three times. The leaves were cut into 0.5 cm fragments, and then transferred in 50 mL centrifuge tube which was contained 15 mL deionized water. After the fragments were shaken for 24 h at room temperature, the initial conductivity (EL_1_) was measured with a conductivity meter. The solution was then autoclaved at 121 °C for 10 min, the second conductivity (EL_2_) was detected after the solution was cooled to room temperature. The ratio of EL_1_/EL_2_ × 100 was the relative EL.

#### 5.3.2. Ion Content

Total of 0.1 g leaves were dried in the oven and milled into fine powder. The powder was then solubilized in 5 mL nitric acid and 1 mL H_2_O_2_ with microwave digestion (Ethos One, Italy). The procedure was set as follows: 130 °C, 12 min; 160 °C, 38 min. After that, the ion contents, including K^+^ and Na^+^, were detected with inductively coupled plasma mass spectrometer (ICP-MS, X Series 2, Germany).

#### 5.3.3. Chlorophyll Content

The chlorophyll content measurement was according to Hu et al. (2012) [6]. To extract the chlorophyll, a total of 0.1 g leaves was submerged into 10 mL dimethylsulfoxide (DMSO), which was contained in 15 mL centrifuge tubes. The tubes were incubated in the dark for 48 h until the leaves were totally faded. The absorbance at 645 nm and 663 nm of the extract solution was detected with the spectrophotometer (UV-2600, UNICO, Shanghai, China). The absorbance of DMSO was used as a blank. The chlorophyll content was calculated using the following formula:

Chl-a content (mg·L^−1^) = 12.7 × OD663 − 2.59 × OD645, (1)

Chl-b content (mg·L^−1^) = 22.9 × OD645 − 4.67 × OD663, (2)

OD645 and OD663 were the absorbance of the extract solution at 645 nm and 663 nm, respectively. Chl a/b is the ratio of Chl-a/Chl-b.

#### 5.3.4. Chlorophyll (Chl) *a* Fluorescence Transient

Chlorophyll *a* fluorescence was measured after 7 days of salt stress treatment with a pulse-amplitude modulation fluorometer (PAM-2500, Heinz Walz GmbH) according to Chen et al. (2013) [70] with some modification. The plants were exposed to the dark for 30 min to ensure sufficient closure of PSII reaction centers. The third topmost expanded functional leaf was selected to be measured. The intensity of the saturating light pulse was 3000 μmol photons m^−2^ s^−1^. The Chl *a* fluorescence transient was then detected and digitized between 10 μs and 310 ms under room temperature.

#### 5.3.5. The JIP-Test

The JIP-test was based on the theory of energy fluxes in bio-membranes and employed to the multi-parametric analysis of Chl a fluorescence transient [70]. For JIP-test, F_O_ (fluorescence intensity at 20 μs); F_M_ (the maximal fluorescence intensity); F_J_ (fluorescence intensity at 1.96 ms); F_I_ (fluorescence intensity at 23.4 ms) were extracted from the original measurements. In addition, F_300μs_ (fluorescence intensity at 300 μs) was also extracted for calculation of M_O_ (the initial slope of the fluorescence kinetics). According to Srtasser et al. (2010) [14], the parameters for JIP-test include: (i) the specific energy fluxes per reaction center (RC), such as absorption (ABS/RC), trapping (TR_O_/RC), electron transport (ET_O_/RC), electron flux reducing end electron acceptors at the PSI acceptor side (RE_O_/RC) and dissipation at the antenna chlorophyll level (DI_O_/RC); (ii) the quantum yields and efficiencies, such as maximum quantum yield for primary photochemistry (φP_O_), the efficiency that an electron moved further than Q_A_^−^ (ψE_O_), the efficiency that an electron was transferred from intersystem carriers to reduce end acceptors (δR_O_), the quantum yield for end electron acceptors reduction (φR_O_), probability that a PSII Chl functions as RC (γRC); (iii) performance indexes based on energy conservation such as PI_ABS_ and PI_total_.

#### 5.3.6. RNA Extraction and Gene Expression Analysis

For RNA extraction, 0.1 g bermudagrass leaves were sampled after 12 h treatment, and Trizol reagent (Invitrogen, Carlsbad, CA, USA) was used to extract the total RNA [67]. The RNA was then reversed to cDNA by M-MLV reverse transcriptase (Promega, Madison, WI) and oligo (dT) primer according to the operation manual. The gene expression was analyzed by two-step RT-PCR procedure with StepOnePlus Real-Time PCR Systems (Applied Biosystems, USA). SYBR Green (Toyobo, Osaka, Japan) was used as fluorescent dye and Real-time PCR Master Mix (Toyobo) was used for the reaction according to the operation manual. To quantify the gene expression, the Actin gene of bermudagrass was used as internal control, and the comparative Ct method was used to calculate the relative quantity of the target gene [71]. The primers of the specific genes are listed in Table 1.

### 5.4. Statistical Analysis

The statistical analysis used in this study was based on one-way analysis of variance (ANOVA), Duncan’s multiple range test. The statistically significant was considered when *p* < 0.05. Data were showed as mean ± standard deviation.

## Figures and Tables

**Table 1:** Primers of candidate genes in bermudagrass.

Gene Name	Primer Direction	Primer Sequences (5′-3′)
*ECA4*	F	GCCTTCGTCGAGCCGCTCGT
	R	GCTGATAAGCTGGAGCACGC
*RAN1*	F	AATGCAGAAGGAAAATATTT
	R	TCCTACTTTGGTCGCTTGTA
*MHX1*	F	ATGGCGAGCACTGCTCTGTC
	R	GTAGTTCCACACCTTCTCAT
*psbA*	F	AACAAGCCTTCTATTATCTATT
	R	GGGCAGCGATGAAGGCGATA
*psbB*	F	TGGGTTTACCTTGGTATCGT
	R	TTCCTCCTGAAATACTCCAA
*psbP*	F	AGGTGTACAAAGATGTGATT
	R	GCACCCAGTGCATGTCTGGT
*psbY*	F	TCTCATCTTCCTTCTCCACT
	R	CTTCCATGGAAGCAGGACTT
*Actin*	F	TCTGAAGGGTAAGTAGAGTAG
	R	ACTCAGCACATTCCAGCAGAT

F = forward; R = reverse.

## Supplementary Materials

The following are available online at https://www.mdpi.com/2223-7747/8/9/347/s1, Figure S1: O, J, I, P steps of bermudagrass under different treatments, Table S1: parameters of JIP test analysis in bermudagrass under different treatments.
