# Supplementary material for: Salt-Induced Damage is Alleviated by Short-Term Pre-Cold Treatment in Bermudagrass (Cynodon dactylon)
Source: Plants (Basel). 2019 Sep 13;8(9):347. doi: 10.3390/plants8090347 (PMC6784090; doi:10.3390/plants8090347)
Supplement: Supplementary file 1 [file plants-08-00347-s001.zip › supplementary material/primer.docx]

comp33654_c0 psbA photosystem II PsbA protein

AACAAGCCTTCTATTATCTATTTTCTTTCTAGTTAATATGTGTGCTTGGGAGTCCTTGCA

ATTTGAATAAACCAAGATCTTACCATGACTGCAATTTTAGAGAGACGCGAAAGTACAAGC

CTGTGGGGTCGCTTCTGCAACTGGATAACTAGCACCGAAAATCGTCTTTACATCGGATGG

TTTGGTGTTTTGATGATCCCTACCTTATTGACCGCAACTTCCGTATTTATTATCGCCTTC

ATCGCTGCCCCTCCAGTAGATATTGATGGTATTCGTGAGCCTGTTTCTGGTTCTTTACTT

TATGGAAACAATATTATCTCTGGTGCCATTATTCCTACTTCTGCGGCGATCGGATTGCAT

TTTTACCCAATTTGGGAAGCTGCATCTGTTGATGAATGGTTATACAATGGTGGTCCTTAT

GAGCTAATCGTTCTACACTTCTTACTTGGTGTAGCTTGTTATATGGGTCGTGAGTGGGAA

CTTAGTTTCCGTCTGGGTATGCGTCCTTGGATTGCTGTTGCATATTCAGCTCCTGTTGCA

GCTGCTACTGCTGTTTTCTTGATTTACCCTATTGGTCAAGGAAGTTTCTCTGATGGTATG

CCTTTAGGAATATCTGGTACTTTCAACTTTATGATTGTATTCCAGGCAGAGCACAACATC

CTTATGCATCCATTTCACATGTTAGGTGTAGCTGGTGTATTCGGCGGTTCCCTATTCAGT

GCTATGCATGGTTCCTTGGTAACCTCTAGTTTGATCAGGGAAACCACTGAAAATGAATCT

GCTAATGAGGGTTACAAATTTGGTCAAGAGGAAGAGACTTATAACATTGTGGCTGCTCAC

GGTTATTTTGGTCGATTAATCTTCCAATATGCTAGTTTCAACAACTCTCGTTCTTTACAC

TTCTTCTTGGCTGCTTGGCCTGTAGTAGGGATCTGGTTCACTGCTTTAGGTATTAGTACT

ATGGCATTCAACCTAAATGGTTTCAATTTCAACCAATCTGTAGTTGATAGCCAAGGTCGC

GTTATTAATACTTGGGCTGATATCATCAACCGCGCTAATCTTGGTATGGAAGTAATGCAC

GAACGTAATGCTCACAACTTCCCTCTAGACTTAGCTGCTCTTGAAGTTCCATCTCTTAAT

GGATAAGGTTTTTCTACTAACATATAGGAATTTTTAAAGGGAGGAAAGCCAGAAATACCC

AATATCTTGTTTCAACAGGATATTG

**Primer:**

**F:** AACAAGCCTTCTATTATCTATT

**R:** GGGCAGCGATGAAGGCGATA

comp17393_c0 psbB photosystem II PsbB protein

CTTGTCATTTGGGATCATTTCCCATATAAAAAAAGAGTCAAAGAAAGGACAGGACAGGAA

ATGTTTCCCATATCAAATCAAAAAAGAGAGGCGAAAGATACCGTAGCAATGTGGTATCAA

ATTGTCTGTCTCCTTGTAGTTGGATCCCCAACTTTTTGGAATGTTCCAAATTCCACTTGA

GCATCCAAGTCTGGATCAATACCAGCAAAAACATCTCGGAACAAGGTTCGAGCACCATGC

CAAATGTGTCCGAAAAAGAAGAGCAAAGCAAAGGTAGCATGACCAAAAGTGAACCAACCC

CTTGGACTGCTGCGAAAAACACCATCTGATTTCAAAGTAGCTCGATCTAATTCAAAAATT

TCTCCTAATTGGGCACGCCTCGCGTATTTTTTTACAGTAGCAGGATCAGAATAACTTACT

CCATTAAGTTCGCCACCATAGAACTCCACCGTTACGCCTACTTGTTCAACGCTATATTTG

GATTCTGCTCTTCTAAAAGGAACGTCCGCTCTCACAATTCCCTCTTCATCTACCAAAACT

ACCGGAAATGTTTCAAAAAAAGTAGGCATACGACGTACAAAAAGCTCCCGCCCTTCTTTA

TCTCTAAAGACGGGATGTCCTAACCATCCAACAGCTATTCCATCCCCATTGTCCATTGAG

CCTGCTCTGAATAATCCCCCCTTTGCCGGATTATTACCAATATAATCATAAAAAGCTAAT

TTTTCGGGAATTTTAGACCAAGCTTCTGATAAACTAAGATTTTCGGCTAACCCATCACTA

ACTCTTCGATATATTTCTTGCTGAAAGTATCCCTGATCCCACTGATAACGAGTAGGTCCA

AATAATTCGATTGGGGTAGTTGCCGATCCATACCACATAGTTCCAGCAACTACGAAAGCT

GCAAAAAAAACAGCAGCGATACTACTGGAAAGTACAGTTTCAATATTGCCCATACGTAAT

CCTTTGTATAGACGTTGAGGCGGACGGACACTAAGATGGAATAGGCCCGCTAATATGCCC

AGTGTACCCGCAGCAATATGATGCGAAGCTATTCCCCCCGGAACGAAAGGATCAAAACCT

TCTGCACCCCACGCAGGATTTACAGCTTGTACTTTTCCAGTTAGTCCATAAGGATCGGAC

ACCCATATCCCAGGACCATACAAACCGGTTACATGAAATGCACCAAAGCCAAAACAAGCC

ACCCCTGCAAGAAATAAATGAATTCCAAAGATCTTGGGCAAATCTAAAGAAGGTTTTCCC

GTCCGCTCATCACAGAATATTTCTAGGTCCCAATATACCCAATGCCAGATAGCTGCCAAG

AAACACAAGCCAGAAAACACAATATGCGCACCTGCCACACCTTCATAACTCCAAATACCC

GGATTCGTTATAGTTCCTCCTGAAATACTCCAACCACCCCACGAATTGGTTATTCCTAAA

CGAGTCATGAAGGGGATGACGAACATACCTTGTCTCCACATTGGATCCAGAACAGGATCA

GAGGGATCAAAAACCGCTAATTCGTATAAAGCCATCGAGCCAGCCCAACCAGAAACTAGA

GCTGTATGCATTATATGCACCGAAAGCAATCGACCCGGATCATTCAATACGACAGTATGA

ACACGATACCAAGGTAAACCCATGGAAATACCCCTTTAGCAAAGAAAAATAGACACGATG

TCGTTTTATTTTATCGCATTGGAAAAGACTATCCATATCCTATGTACCTAACCCTTCTAG

GGGATTCTGTGTCAGAAAGCGTGAATATTTATTTCATTCCATTAAAAATAAGAAGCCAAT

TCTGTTTGTAAGAAGAAAGAGAAGCAGATCTATTCTATACTCGATAAGTGCCAATATGCA

ATGGGTAGCTTGGGCTATTTTGAAAAACGAAGAATAGATCCCTAATCCCTCTTTGTTTAT

CATTCGAATCACAGATTCCTTTTTCTTTATTCAGTCTGTCTTATCTTCCTTATATGTATA

ATTTTGCAATCTATGTATTATTTCAATCTATGTATTAATAGAATCTATAGTATTCTTATA

GAATAAGAAAAAAATGAAGATAATAAACTGCGGATTCTTTCTTTCTCTTCCATTCTTTAA

GTTTCCATATTAAAGTGTAGTTTTCTTACTTAAATCTAATAATATTAATCTAATATGCCC

ATTGGTGTTCCAAAAGTACCTTACCGGATTCCCGGAGATGAAGAAGCGACTTGGGTTGAC

TTATACAATGTTATGTATCGAGAAAGGACACTTTTTTTAGGTCAAGAGATTCGTTGCGAG

ATCACGAATCATATTACAGGTCTCATGGTATATCTCAGTATAGAAGATGGAATTAGCGAT

ATTTTTTTGTTTATAAACTCCCCTGGCGGGTGGCTAATCTCAGGAATGGCAATTTTTGAT

ACGATGCAAACGGTGACACCAGATATATATACAATATGCCTCGGAATAGCCGCGTCCATG

GCCTCCTTCATTCTGCTTGGAGGAGAACCTACTAAACGTATAGCATTCCCTCACGCTAGA

ATTATGCTTCACCAACCGGCTAGTGCTTATTATCGGGCAAGGACACCAGAATTTTTACTA

GAAGTGGAAGAGTTACACAAAGTTCGCGAAATGATCACACGGGTTTATGCACTAAGAACA

GGCAAGCCTTTTTGGGTTGTATCCGAAGACATGGAGAGGGATGTTTTTATGTCAGCAGAC

GAAGCTAAAGCTTATGGACTTGTTGATATTGTAGGGGATGAAATGATTGACGAGCACTGC

GATACTGATCCTGTGTGGTTTCCAGAAATGTTTAAGGATTGGTAGTGGCTGAATTTCTTG

TAAATTTATTTCAAATCTAAATTTTGGTTTTATGCGATTTTATAGAAAATAGAAATACTT

CATGATGATGAATCATCAGGTTAAGATCGATCTAAACCAATCCATTTTTTTCTATATACA

TACGACATGCCAACAGTTAAACAACTTATTAGAAATGCAAGACAGCCAATACGAAATGCT

AGAAAAACGCCCGCGCTTAAGGGATGTCCTCAGCGTCGAGGAACATGTGCTAGGGTGTAT

ACTATCAACCCCAAAAAACCCAACTCTGCCTTACGTAAAGTTGCCAGAGTACGATTAACC

TCTGGATTTGAAATCACTGCTTATATACCTGGTATTGGCCATAATTTACAAGAACATTCT

GTAGTATTAGTAAGAGGAGGAAGGGTTAAGGATTTACCCGGTGTGAGATATCGCATTATT

CGAGGAACCCTAGATGCTGTCGCAGTAAAGAATCGTCAACAAGGGCGTTCTAGTGCGTTG

TAGATTCTTATCCAAGACTTGTATCATTTGATGATGCCATGTGAATCGCTAGAAACATGT

GAAGTGTATGGCTAACCCAATAACGAAAGTTTCGTAAGGGGACTGGAGCAGGCTACCATG

AGACAAAAGATCTTCTTTCTAAAGAGATTTGATTCGGAACTCTTATATGTCCAAGGTCAA

TATGGAAATTCTTTCAGAGGTTTTCCCTTACTTTGTCCGTGTCAACAAACAATTCGAAAT

ACCTCGACTTTTTCAGAACAGGTCCGAGTCAAATAGCAATGATTCGAAGCACTTCTTTTT

CCATTACACTATTTCGGAAACCTAAGGACTCGATCGTATGGATATGGAAAATACAGGATT

TCCGATCCTAGCGGGAAAAGGAGGGAAACGGATACTCAATTTAAAGTGAGTAAACAGACT

TCCATACTCGATCTCATAGATCCCTATCGAATTCTGTGGAAAGCCGTATTCGATGAAAGT

CGTATGTACGGCTTGGAGGGAGATCTTTCCTATCTTTCGAGATCCACCCTACAATATGGG

GTCAAAAAGCCAAAAAAATAAGTGATTCGTTTTTAGCCCTTATAAAAAGAAAACGGATTC

TTGAACCTCTTTCACGCTCATGTCACGTCGAGGTACTGCAGAAAAAAGAACTGCAAAATC

CGATCCAATTTTTCGTAATCGATTAGTTAACATGGTGGTTAACCGTATTATGAAAGACGG

AAAAAAATCATTGGCTTATCAAATTCTCTATCGAGCCGTGAAAAAGATTCAACAAAAGAC

AGAAACAAATCCACTATTGGTTTTACGTCAAGCAATACGTAGAGTAACTCCCAATATAGG

AGTAAAAACAAGACGTAATAAAAAAGGATCGACGCGGAAAGTTCCGATTGAAATAGGATC

TAAACAAGGAAGAGCACTTGCCATTCGTTGGTTATTAGAAGCATCCCAAAAGCGTCCGGG

TCGAAATATGGCTTTCAAATTAAGTTCCGAATTAGTAGATGCTGCCAAAGGGAGTGGGGG

TGCCATACGCAAAAAGGAAGCGACTCATAGAATGGCAGAGGCAAATAGAGCTCTTGCACA

TTTTCGTTAATCCATGAACAGAATCTAGGTATGTAGACACATGGATCCATACATCTCGAT

CGGAAAAGAATCAATAGAAGGAGAATCGGACGATATCTTTTTCGAAACAAACAAAAAGGA

AAAGAAAGAGAAAACAGAAATCATGATCAACTAAGCCCTCTCGGGGGCTTGCTTAAGAAT

AAGAAAGAGGAATCTTATGGAAATAGCATGGAATAAGGTTTGATCCTATTCATGGTGATT

CCGTAAATATCCCATTCCTAAAATCGAAATAATCGGGACTTTTCGGAGATTGGATGCAGT

TACTAATTCATGATCTGGCATGTACAGAATGAAAACTTCATTCTCGATTCTACGAGAATT

TTTATGAAAGCGTTTCATTTGCTTCTCTTCAATGGAAGTTTCATTTTCCCAGAATGTATC

CTAATTTTTGGCCTTATTCTTCTTCTGATGATCGATTTAACCTCTGATCAAAAAGATAGA

CCTTGGTTCTATTTCATCTCTTCAACAAGTTTAGTAATAAGCATAACGGCCCTATTGTTC

CGATGGAGAGAAGAACCTATAATTAGCTTTTCGGGAAATTTCCAAACGAACAATTTCAAC

GAAATCTTTCAATTTCTTATTTTATTATGTTCAACTTTATGTATTCCTCTATCCGTAGAG

TACATTGAATGTACAGAAATGGCTATAACAGAGTTTCTGTTATTCATATTAACAGCTACT

CTAGGGGGAATGTTTTTATGTGGTGCTAACGATTTAATAACTATCTTTGTAGCTCTAGAA

TGTTTCAGTTTATGTTCCTACCTATTGTCTGGATATACCAAGAGAGATCTACGGTCTAAT

GAGGCTACTATGAAATATTTACTCATGGGTGGGGCAAGCTCTTCTATTCTGGTTTATGGT

TTCTCTTGGCTATATGGTTTATCTGGGGGGGAGATCGAGCTTCAAGAAATTGTGAATGGT

CTTATCAATACACAAATGTATAACTCCCCAGGAATTTCAATTGCGCTTATATTCATCACT

GTAGGACTTGGGTTCAAGCTTTCCCTAGCCCCTTTTCATCAATGGACTCCTGACGTCTAC

GAAGGAGTGTGGTTCGTTCGACAAATTCCTACCTCTATATCTATCTCTGAGGTGTTTGGG

TTTTGCAAAACTCCATAGACATGCAGAAGAGAAATGCTATCCCCACTCCGACCAAGACAG

AACTTTTACCAAAAGTTTATTGTGATCTTTTTGTTCAAATAACAATTAAGGTGAAGCAGG

GTCAGGAACAACGAATCTCTTTATGATAAACAGATCCATTTTGCAAGCTCGTTATTACGG

GTAGTTCCTACAAAGAATCGGACTAATGACGTATACAATGCTTGAATTATCGATGTAGAT

GCTACATAGTGGGTTCTCATCCTTCAGAGACTACGAGTGTAATAGGAGCATCCGTTGACA

AAAGGATCACCCTAAGATGATCATCTCATGGCTATTGGGAACGAATCAAATCAGATGGTT

CTATTTCTCAACCTTTCTGACTTGCTCCTACGGAACCAAGGTCGAAAGGATTGAAAAAGT

CAGTCATTCACAACCACTGATGAAGGATTCCTCGAAAAGTTAAGGATTAGTA

**Primer:**

**F:** TGGGTTTACCTTGGTATCGT

**R:** TTCCTCCTGAAATACTCCAA

comp32891_c0 PSBY photosystem II PsbY protein

AGTCTGAGACACGGCTGGTTTATTCCCAACATCTGAGCTGGGATATGGTGATTTACAATG

GCCCATCCTGTGTATGTATCCTTGAGCCCAAAACAATGAAGTAATCATAAGTTACAGACA

TGCGGTGAATCCAAATGTGCTACTACAAAATTGACAACTCAAAAGAATACCAAAACTTTT

TGCACACCGCGCCAATCGCTTTTCACCTTACGGCAATCTGATCCAAAGTCTCTTAAACAT

CAACATAAAGCTAAAATCGATGGTCTTTCCCTTAGCATACCTTGCTTACAGGTGACAGAC

GAACCATTGGAACCTGGAGCGTGCAGGACTGAGTTTTAACTCACACACTCATCAGATGCT

GAATAGCTCTTTTCAAGGATGAATTTAGGCTCGCTAGGATAGACAAAGACAGTAGCTGCC

GAGGTGGAAGGAGTCAATGCGGCAGAATACCTCACCGAAGTTACTGTGATCTTTGGCAAC

TGCTGCGAGCCAGAACCAAGCGGCACAAGCTTATGACTGATAACATGCTCCGATTTCGGT

AGAACGAATGCAGCGTGATTGTGAGCCCCAGAAAAAACGAAGTTCTGGGAATCAACAAGG

GAGTACTTAATTTCCTGAAGTAAGGACGTTGAATTGTGGATCTTCACATAAAAAGTGAAA

GGGATTCCAAGGATAGCGTAGGGAGGGCACTCCATACTCACAACAAGCGGTGGCTCCTCA

ATATTGACCTCAGGTAACCTTTGTTTCATGATAACACGGCTCTCTTGACCTTCTCCGAGG

CTTGAATCCCTTGACCAGTTCAGACAAATTTCACCTAAATTGAAATTCGAACTGACTGCC

TGTGGGCGAACAGAAAATATCGCTTTGTATTCTTCATTGGGAGCAATAACTGCATGTCCA

CTGGTAATCCCGCTGACCTGTTGCACTGAGCAGAGCTGCTTCCCATCACCATCAGGCTCG

ATGGTCATCGAATGCAAATGCAAAGGCACCTCAGCGCAGTTCCTAGCATTCACAACAAGC

ATATTCGACTCATTCATAGCAAGAGAACATATTTTATCATCACCACTTGACGTTCTGATC

CCCGATAGCAATAGGGGTTCTCGCCTAAATGGTCTCAAAAACTGGTGGCTAACTGTCAAC

GGGATCTTCCCGTCGATTTGCAAACTTCTATGCACGTTGAGTCTGTGCAGTACTGCTTCT

TCGCTTGGGCCTAGGGAGTAACCTAGTGAAACATAAAGCATAACCGACTTTGCTCGATGC

CATTTGATTTCTAATTTGCATGACCAAGAATCACCCACACTCAATTTGGGAACAGAAAGA

ACCCCAAATGAGTATTGGATCTTTTTTATACTATCAACCTCTTCCTTTGACTCTTTGTCC

TCGGACACTGCTGAGACCCCAAGAAGTTCAACATGGTGACTTTCAGATTCCTCTGCTTCC

CTTGGACTCATCAGCAGACCACCACCTCGGGCGTCAACAAGGTTAATTTTCAGTTCACCA

GAATGAACTGCGTGCCCTTTACATAGTATGGTTACTGGTACAACAAATAACTCTCCAACT

AATGCAGGGCCAGCAGAATTTAGGTCAAGGTCAACTTGAGCATCTGGCTCTTCAACTTGG

ACCAGTTTCTGCCCGGAAAGAGCAAGAACATGATCCTTCATAGGCAGTGTCTCCACTTGG

TCCTCAAATTTCCATAATGGGAAGTCTTCCATGGAAGCAGGACTTTCAGCTTGGCAGCAA

ATCACAAGGTGTTTATTTATCGTTGCCTTCACAGATAAACATTCTAGCTTTCCACTTTGT

TCTGATTTAATTTCATGTGTCAACCGCATCCATTTGTTGGTGAATAGTGTAAGAGAAGTA

GAATCTTCCTGTGCGCTGTGTATTACAAAGTTACAGTCTGATTGATTAAACTGAACCTCC

AACTGATCAATCGTGAGTGGAGAAGGAAGATGAGATAACAGTGACACACTGACAAGCACA

GGTGAACCAGGTTTTACAGATTGATCATGAAACGCAACAGATGCAGTAAGCACTATTCTT

AGTGGACTTATTTGATCAATATCAAGGTGAGTGCATTCTTCAATAGCGTTGTTAAATGCA

TCATCAGTTCCTTCTGAGTGCTTTCCTTCCAAAACGTTAATAACTTCCTGCAGTATGGTC

TCCCTCCTCAAAATGGCAGGAGAGCCCGCAGGTCCA

**Primer:**

**F:** TCTCATCTTCCTTCTCCACT

**R:** CTTCCATGGAAGCAGGACTT

comp30095_c0 PPL1 photosystem II oxygen-evolving complex 23kDa protein

CATTCATTTGCGTACTGCGGTGTTTCATTTATGAAAATGAAGCATTGAGCAAACATACAT

GTTTCCCACAAGAGACGTCTTTCTATGGCTTACAGATTACAAACATAGGTTTGGATATCC

TTGGTTCAAGGATATCAGTATGTCAGTAACCACCATGATATATATACCAAGTACCAACAA

ATGTCCCAAAAACGTATGCCCCAATATAGAACAGATCAGTATAATTCACATTCCCGATTA

CTATTGAACCTCAACATTACAGCTCCAAAAGTGAACAAATCAACAAAGATCATGAGAAGG

CCTCAGACATCCCTCAAATTCTGTTTTCAATTCTAAAGGAATCAATAACGGTATGCAGCC

TATCCTTCATTTTGTCCCATCTCCTCTCATTTGCTCCAGTAGTCAGTGTGTAAAATTTGC

CATTTCCAATAGCAATTGCACCCAGTGCATGTCTGGTGTAATTCGGAGCCTGAGCTGTGA

ACTCAAAAGTGTAGTAAGCTCTTCCGTCAACCTCATTCTCTTTTGCCTCAATTAACTTTG

TCTTTTGATTCGGTGGAGCTAAAACCTTTTGTATCAATGTTTCAGCAACCTTGTCAGGAG

GACCAAGCTCACGAATATCTTCCTTGCTGGTGGCAATCGTGTTGAGGCTGACACTCTCCA

GTGGCTCAATCACATCTTTGTACACCTTGTCTTGCCCTTGCACGGCCACTTCCTGGACAC

CTCCAACCTCAGCTTGGCACAAACATAAATTTGTGATTCTGTTCAAGTACACTGCGTAGA

CTGTTGCTCACCTGCCATCCGAATGGATAGAGGAAGGAGTAGCCAGTCTTCTTGTCGATG

ACAGGCAGGAACCCTTTCTTAGCCTCTGCTGCGAATGCTGCCCGATCAGGCCGAGACAGA

AATGTCGCGACCGCCCCCGCCACGAGCACCTGCCGGCGCCCTGAGCCCTCCTCCTCCTCC

ATCTCGTTGTCGAGCTGTCGGGGAGGCGGAGCACTGCAGGCGGCGGCGACTGACCTCAAG

ACGCAATGCCGTTTGCGGAGAGAAGGGATTGCCGGACGACGCGCGGTGTTGGCGGGGGAG

GAGGCGGCCCGAGGGAGCGGGAGGGAGGCGGCGGGCACGGCCGTGGCCATGGCGTGGACG

AGGATAGCGAGGAGGCCAAGCTTGCGGAGGAGGGACGAGACTTGGATTTGGCTTCAGTTC

TGGTCCACACACACGAGGGCGAGCTTGCACCTCAAAGCGGCCCATGTTTGCCTAACATTT

CCACCGTAGCAGATGCGCATCATCAAGTGACCAGTCCTCTGGTTCTCAGAAAAAAAAAAT

TTACTGGATCAATTTCCCTTATCAATCAACAAACAAAAAGTACTACAGTATAGTACTGAG

ATCGATTTGAACGAATTGGAAAACTGATGAGAGGCCACCGATTGAACCCTCGTGTCACGA

TTATTTACTGTATAACGACCATACATGCGTACTACACACGATGAATTAATGGGTCAAAAT

AAAACACGAAAGGTAATTCAGCACTGGAGCTTGGAGCCGTGGTCCTGGTGCAGCGCCGAG

ACTGATCACCCGCCACGAACACTCGCCATAGCGAGGGAGAGACCACTCAGCAGGATCAGA

TACAACCCGAAGGCCACCGGCGGGCTGGCGGTGCCGGCGACGATTCCGGTGATAGCTGAC

GATGCGACTGTCATTCCAACGACAGCGACGGATGAAAGGTTGCCACCGCCACGATGGCCA

TCCTTGATGCTTCCAGTTTCTGCACGCTCTGCCCTGTTGGCCTCATCAGCAGCAGCAATT

GCACCGCCACCAGCTTCCGCACCCGTTGCCTCGTCACTCTGCTCGGCAGACAGCAACATC

AGCGCCAATTTTACATTACAGTATCATGACTCAATGGAAAGAACATAGTAGACCGAGTCA

TCATCGATCGAGACGTTCGGGCGTCGGGACCTGGAACACTCCGTGGGTGTGGACGACAAC

GGCGGCGCTGTCGGAGTTGGCGAAGTTGACGGGCGGGCGCTGCGTCTCCGGCAGCCAGTC

GTGCACGACGGTGGAGATGAGCTGGTCCGCCATGATGAATAGTAATCGACAAGGAAACCG

ACGGGCACTCACTTCGCTCGGGCTTTGTGCACCAC

**Primer:**

**F:** AGGTGTACAAAGATGTGATT

**R:** GCACCCAGTGCATGTCTGGT

comp32660_c0 RAN1 Cu2+-exporting ATPase

AAATTCAGTTCGTCGGTTCTGCCATGAAGGCTAAGATTGATGCAGTCACTTGATCTAATA

TGAGAGTAAGAGTTAATGTGCAATGCAATTCAATTGCAAAACTGTAGGATATGTACAAGA

CTTTGACAGCCAAAATTGCAATATAGACAAGGGAGACACATCTTTCACATCTCCCGTAAA

TACACACGACAGAATGCCACAAAAGGATATCACTCCACTGTTATCTGCAGAACAGTTGTA

AGCCTAGGTTTTCTATACCTCCTCAGCAGCAGCGAGGAGCACACAACACTAACTGACGAG

AACGCCATGCACAATCCCGCCAGCCACGGCGGCATCTGGAGTCCCGTGAATGGGAACAGC

GCACCTGCAGCCACTGGGATAGCCACAATGTTGTACGCCATGGCAAAGAAGTAGTTCCAC

CGGATCCGGCTGAATGTCTTCCGGGAGAGGTCGATTGCCGTGATCACGTCCTCCAGGTTG

TTCCGGACCAGCACGTAGTCTGCTGCCTCAATGGCGATGTCCGTTCCGGCACCAATAGCC

ATCCCAACGTCAGCGGCAGCTAGGGCTGGGGAGTCGTTGATGCCATCTCCAACCATCGCA

ACAACGCTGCCATCCTTTTGGAGGGAACGGATGACATCGGCTTTTCCAGCTGGCATGACC

TCTGCTCTCACATCCTCAATGCCAACCTACAGAATCACAATATTACCAAGAGGTAACTAA

TAGATGAGATGCAGATGGTTAGCTTAAACATGACAATTCAAACTGACCTCATTTGCAACT

GCTTGTGCAGTCCTTCGGTTGTCCCCTGTGACCATAACTGGACGAACACCCATCTTTTTG

AGGCCCTCTACAACCACAGCAGCCTCTCTTTTCAAGGGATCAGTGATCCCAATCAAACCA

ATGAATTCACCATCATATGCTACAAGAATGCCAGTTTTTGCTTTCATCTCCATGTCTATC

AAGAAGTTTTCAGCTTCTTCAGGAATATTTGTCCCATTCTCAGTTATCAAAGCACGGTTT

CCTACCAAAATTTTCTTCCCATTGATCCAGCACTGAATCCCTTTGCCTGGTAAGGCAGAA

AAGTCTGCAGCATCCAGGAGCCATTGAGAAAATAGCTCTTCCTTATTTTTCTTCATGTTG

TCTTTTGATGAGGGGAGTTTGCCAAAAAAATGGAAATGAAATGCATAATCCAAGATAGCT

TTTGCAAGTGGATGCTCGCTGCTTGCCTCTGCAGATGCTACCAATGTGAGGAAGTCCCCT

AGCTCCATTCCAGAAAAGGTCTTCGTTGTTGTTACAGTAGCCTTTCCTTGGGTCAGTGTC

CCCGTTTTATCAAAGATAACATATTTTACATTCTGGGCTCTCTCCAGAGCATCTCCACCC

TTCACAAGTACTCCATGGTTAGCCCCAACTCCGGTTGCAACCATAACAGCAGTAGGTGTA

GCCAGACCAAGAGCACACGGGCAGGCAATCACAACAACAGATATGGAGAACATGAGAGAG

AAAACAAAGCAGTTGCTAGTTTCAGCAGACCATGAGTTTGGATATGCTCCCAGCGATCCA

CATAAGAACCATGCAAAGAATGTCAGCAAGGACAACGTTATGACAATGGGCACAAAAATG

CTGGCCACATAATCAGCAAATTTCTGGATCGGGGCTTTAGACATCTGGGCAGTCTCAACC

AGGGATATTATCTGGTTCAAAACTGTCCCTGATCCTACTTTGGTCGCTTGTATATGAAGG

ATGCCATGCAAGTTGACTGTTCCTCCAATTACTAGACTGGATGCTTCCTTTGAGACAGGC

ACAGATTCACCGGTTACCATACTCTCATCAACATGACTTGATCCCCAAATGACAACAGCG

TCTGCAGGAACCTTTGAACCAGGAAGCACTTTTAAGACATCACCAGGTTGCACTAACGAT

GCATCAATCTCCTTCTCATACAAATATTTTCCTTCTGCATTAGAGAACATTGTTTCGAGT

TTATGTAGGTGACTACACTGAAAAATGGGGGCTGAAAAAAAGCCCAAGGGTATGTGAGAA

AGGCTGTGATTCTAACTTCCAAGTGATCCATACGATGTAGCTCAATACCTCTTATATGAC

AACTATGTGGATAAGAACATTCTAGAACCGCTAATCATTACTAATGGAAATGACTTTACA

GTTTGGACCAGGTACCTTTGTCTTTCAGAAGTAAAAGAGCTGTAGCAGGAGCAAGCTCTA

CAAGTTTCTTGATAGCATCTGATGTTTTTCCTTTAGCAAGCACCTCAAGGTATTTCCCAA

ATAGCACAAATGTAATTATCATTGCACTTGTCTCAAAATATAGCGGTGGATGAAATCCAG

TGAGCACCCCATAAAGAAGTGCACAAACAGAGTATACATATGATGCAGTAGTGCCAATAA

CAATTAGTACATCCATATTTGTAGAACCATGTCTCAGGGCCCTATAGGCTGCAATGTAGA

ATCGTTTGCCAACAACAAACTGTACCAAGCTCACCAGAATCCACTTCAGCAAATCTCCAA

TGCGAAATGGTCCACAGTGCATGAGTAGGAATGATCTAATGAAAGGTATGTGCGGGCATA

CCATGCGCATGAAAAATACTGGAGCACTTAGCAACAAGCTAGATCGAAGAAGATGAAGTG

CTCTGGAGGATTCCTGTGCATCATTTGATGCAGCTCGTATATAAGGATTCTGTACATGGG

CTCGAAGTCTGTCGTTACTTTCCAAATTGATGCTATCCACAATCTGTCTCAAACCCACAA

CTTCAGGATCGAATAAAATTTCAACTTCTGAACTTGCAAAATTTACAGCAAAGTGACGCA

ATCCTTCCATTTTCCTCAGGGCATCATGCAATACATCTAAATCTTCCTCAGTATGCAACC

CAGCAACACTTAATAAGACCTTGTCTTGCTCGCTACTTCTTAACAGCGCAGCTTCAAAAC

CTGCATCCTCAATAGCCAGAACAATATCATCTTTGCTAATGGCAGAAGGATCATACTCGA

TTTCCCCTGTTGAAGTTGCCAAAGCAACAGCTACTCTTTTAACACCCGGGAATTTTTTTA

AGATCCCCTCAACTGAGTTCACACACGCAGCACAAGTCATTCCACCTATCCTAAATTGAC

CTGACAATATCTTCTGCGGTTTTGGCTGAGAAACAACAGAGTCCGGGAGAATTTCTGCTT

CGAACCCGGCATCTTCTATTGCTTCTACGATGTCTTCGTCCTTGGCGAGCGCGGGGTCGA

AGACGACGTGGGCGCGGTTCTGGAGCAGCGAAACGTCCACCCGGCGCACGCCCGCCCGCG

CGGAGAGCGCGGCCTCGACGGCGCTGGAGCACGCGGAGCACGTCATCCCGGTGACGCGCA

CCTG

**Primer:**

**F:** AATGCAGAAGGAAAATATTT

**R:** TCCTACTTTGGTCGCTTGTA

comp33103_c0 ECA4 Ca2+ transporting ATPase

GCCATCTGTGACAAGATTGACCCACAGAAGTTGCACAGGGATGAGGCCTTCTGGAATGCC

TATAGCTGATGTCAGGAATATAGATGCTACCTCTCCAATGTTTGAGGAGATCATATATCT

AATAAAAGCCTTCATATTGTTGTAAATAGACCTCCCTTCACCAACTGCTGCAACTATCGT

GCTGAAGTTATCATCTGCAAGCACCATATCTGAAGCTTCTTTGGCAACCTCAGTCCCTGT

AATACCCATTGCTACTCCAATATCAGCCAATTTAAGAGCTGGTGCATCATTCACCCCATC

ACCAGTCATAGCAACCACTTCACCATCTTCTTTGAGCAATCTAACTATCTCCTGCTTGTG

TTTCGGCTCAGCCCTGGAGAAGAGAAGGCCACCCTGTTGCCTTAAGAGCTTCTTTTTGTC

AGAATATGCCATGAATTCCTTTCCTGTGAAGCTCTTTGAACTGATATCTTCGTCATGACT

GAAAACCCCTATTTCACGGCATATCGCCTCTGCTGTTTCTTTGTTATCTCCTGTTATCAC

CATAACACGTATTCCAGCAGCTCTGCAATCTTCAATTGCTTTGCTTACTTCTTCTCGGGG

AGGATCCCTAAGACCAACAAAACCGCAAAATATCATGTTACTCTCTATGGAAGAGTAGTA

TGAAGGGTCAAGCAGATATTTGTGAGCTGTGTGGTCTTCTCCATCATAGGTTGCAAACTC

TCCCAGATCCTCCTTGTAAGCAAACCCCAAACAGCGCAAAGCACTAGCAGACATTTCATG

CAGTGTAGATAATATGAGTGCTTTGGAACCATCATCCAAGAGCACAACAGATCCATCAAG

CAACTGAATATAACCACATCGCTCCAGTAAATTCTCCACTGCTCCCTTGACAAGCAGCAA

ATTCTTTCCTGAGTCTGCTTTCACAATAACTCCCATTGATTTTCTGGTTCGATCAAATTC

CAGGGTAGCTACTCTCTTAGCAGCATTGCTCCACCACTGACAGCATCTTAGCAAATCAGA

CGAATCCAGAGATGGTGTATAGCCACCAGGAAGCCCCATTTTCTCAACCATCACCTTCAA

AGCAGCTTCTGTGGGCATCCCAGTAGCAACATACTGGTGCTCAGAGTGGGCAATGCTTGC

ATCGTTGCAAACTGCAGCAATCTTTGCAATCATCTGCAGATTTTCATCCATGGTCGAGCT

TGGCCAATCATGTATCTGACCATCAGTTGGATCATACGTTGTCCCATCGACCTTAAAGCT

CCTAAGTGTATCAGGCCACCTCCCAATTGCCACAAGCCTCACAGCTGACATTTGGTTGGT

GGTCAAAGTTCCGGTCTTATCCGAGCAAATCACAGTTGTACAACCCAACGTCTCCACACT

GGGCAACTTTCTCACAAGAGCATTCTTCTGTGCCATCTTCCTTGTACCAAGTGCCAAGCA

CGTGGTGATCACAGCAGGCAAGCCCTCTGGAATCGCAGCAACAGCCAGTGCCACTGCAAT

CTCAAAGTAATACGTGCACTTCTCAAATGAGAACTTGAAATTTCTAGGCCATCCGTCCAC

ATACTCCCACGTCAGGAAATACTTCACGTTGATGAGCCATACCAAGGCGCAGATGACACC

AATTATGGCAGTTAGTGCCTCACCAAACTCATTGAGCTTCTTCTTCAGTGGTGTGTCGTC

CTCCTCCTGAGATGCCTCCTGGATCTGGGCATGGATCTTGCCTATTTCGGTTGCCATGCC

AGTGCCTACCACAACACAGACTGCACTGCCGTTGACGACAGTGGTGCCGGCAAAAACCAT

GCACTCCTTTCCCTGGATGTCTGTGTCCTCCATGTCGATCTTGTGGCTGGTCTTGTTAAC

CGAAGCCGTCTCGCCGGTGAGGGAGCCCTGCTCGACGCGGAGGGTGGAGCTGATAAGCTG

GAGCACGCGCATATCGGCTGGGACCTTGTCGCCGACGCGGAGCTCGACGATGTCTCCGGG

GACGAGGTCGCGCGCCGGGAGGCCATGGGACCAGCGGCCGTCGCGCTTGACGGTGGCGTG

CTCGGACTGGATCTCCTTGAGCGCCTCGAGCGCCTTCTCGGCGTTGCTCTCCTGCCAGAC

CCCGACGACGGCGTTGACGATGAGGATGAGGAATATGACGAGCGGCTCGACGAAGGCG

**Primer:**

**F:** GCCTTCGTCGAGCCGCTCGT

**R:** GCTGATAAGCTGGAGCACGC

comp32247_c0 MHX1 solute carrier family 8 (sodium/calcium exchanger)

GGTTCCAAAAGAAATAACAAATGCAACATAAATGCATCCATTTTATTAGTAATAGTACAT

AAATAAACTTTACAGCCAGCAAGCACATTTTCATATACATCACACCTTGCTAATAAATGA

ACACCAACTTAATCATATTTACTTTCTAAGGAATATGACTACAGCTGATTTGTTTTAACG

AAAAGTGCTACGTAATTTCCTATACGGTTTACATTATTCCAGAAAATTTTAGGGAAGAGA

GTACAACAAAAACAACCCAAAGCACCATGAAGTATACCGATGTCACCCAAGCCCACAACC

TAGGACCCCCAAGCTCTGCACCAAGTATAATGCGGCGGAGAACCAAAACTGTTATACAGC

CAAACGATGTTGCAAAGAACACTAGAAGGGAGAAGCTGAGACCCTCAGCATTGTCTATGT

ACAGTGGTTCCTGGTAGACAAAGAAGTTGTATACTGTGTCGATCAACCATGGAACACCAA

TGCCGACATATATGTTCACCGAGTTGCTGCAAGTGATGTTGGCTATTGCAGAGTCTGCGG

TGAGTTGACGTTCAGCAGCTATCTTGCTTGCAATTAGATCAGGCCATGAGGTTCCGGCTG

CAAGTGCTGTGAATGCTATAACATATGGACTTATTCCTGGACCAAAAAGTTCCATGTTAG

GGTATGTACAGTCACATCCTAGCGAGGAGATGCATATGAAACTTTGGCAGGAGTATGTTG

CAACCTGTGACGCAGCTTATCTGATCTGTAAGCTTAGTAACGCCATAAGCAATACCACTT

ATGAAGGTTAGCGAGCAAATAAAAGTGATCCAGCCATGTGCAATATGATACGGGGGCACA

AAGGCAAATAGCAATTTCCAAGGCACAATAATCAAGTTCCAAAAAATTTTGGTGAGCCTA

AGGCAGCCAGAGTCCATTTTCCTTGACACAGGGCTCTCCAGCTGCATTTTTTAACAAAAG

CAATTAATACACAAAATGACTGTTAAAAAAATTAGTAACATGCCACATTTTTTTTGTATT

ACTGAAACAATCGTTGAGGGTATCAAAGATTTAGCAAAGCACAAACAGTGGCAGCACAAA

ATCAAATTACCGATACAGCATCAACAAATTGTTGCCACCAAATAAAAAGCCAGGGTGCAT

CATCTCGTTCGTTCTTCGCTGCATCATTCATTGTTGCCGATCCTTCCACATCTTTTTCTG

GGATATTCTGATACTCTATAGAAAAACAGGTATGAATTTCATTCAAAAAATATATCTAAC

ATCATGTACACAGATATGAACAATATGTAAATAGAAGATTCATAATATTCCCCATGTGAA

CAAAGCTTCAAGATTTTTCCAGCGTAATATATCTTAATAAGGCACTCTGGTTGCGATGAA

TAAACTGACGTAATCTGTCACCATCAATCTATCATTATTAAGGACACGGAAACCATACCT

GCCGTATCGTAGGAATGTCCAGTGAATATATCCACAGTATCTTCAATCCTGCCAACATTT

AGAGGGAGTGTCTCGCTAATCTCATCACAGCTGTCATAATCCATCAATGAATTTTCTTCT

GGTACCCAATCTTCAGGCCTCTCACCTCTCACAAAAGGGATTGACACATATGGCCACCGC

TTATCCTGTGCATACGCGTGAAACAGAAGCAATCCATACTGCAAGACTGTCAGCAAGGCC

TCCCTGAGGGTGATCACTCTGGGAGTCCAGACCTGTGCCAGGTGCAAGGTACTTATTATA

CCAAATGCTAGCATAGAAGAACCAAGCATGCAGTTGGCATTGTATAAACTTTTCTACTAA

TATTTAGTGAATATTTAATGCGAGATATGAAAATTATGTCTAAAGTTGTCTCAACAATTA

GAACTGTAATTAAACAGTAGACAAATATTCAAGTCGATGACTTATCGATTTCAACAATTT

CATACTTGTAACCAGAAGAAGCACATTTACCTCTAAGATAATATACAACCAAACGTATGC

CCAGAAAGACCAAAACAGCTCAACTAACCAAACCCCCAAGTCCGAAATCTTCTTCATAGA

GCCAGCCCTTGGCATAATTACACAGACAGCATGTATGGGGAAAAGATCGAATGCAGCAGA

ACCGACAAGGGTACCCGGACCCAAACCTCCAGCTGTCGGTTGACCCAGATTACGGATCGC

GTCGATGGTGGCCAGCGAGATTTGCGGGAAGCTGGTCCCGAAAGCAAGGAGAGCAATGTC

AGCAATAGTGTAGTTCCACACCTTCTCATGCTTCATGACAGGCGTATTCAAGCATGGATC

GATCTTGACCACCTCCCGGGATTGCTTCATAATGCTCTCCATCGATTTAAAGAATCGGGC

AGTGATCGCAGATAAACCAATGAAACAGTATGCGAGGGTCATAGTGTAGATGAATGCACG

GACCCCATTTGAAAGCAGAGTTTCACCATGGAGCACCAGGTAAGTGTCACATGGAGATGA

CAGAGCAGTGCTCGCCATCACAAATGTTGGAAATTAGACAATCACCTCACCCGCAAGATG

AAAGCTTGTAAAATATGTCCACTTCCTGTATGAGCAGTAAGATAAAAAGTCCTCAGGGGA

AAGCAGATCAAAATATTAGATGGCTGCGACAGTTTTTTTTTTCCCACATGCACTGAGCAG

AGACATAATTAACAAAGAAACAAAGAGTTGTTAGTATGACACAGTCCAGCATCAATTAGC

AAGGCAGTGGATCCAAGCATTGACAAGTCTGGACAAGTAAACGATTTAACATCCTGCATA

CAGATTGGGTACAAAACGTGGAATGTTGCCATCATAATCCAGGTATTTATATCATAATAC

CAATAGGGACAATTGGTTACATATACAATTCTGTTATGGGCCCGAGACGAGGTCCCAGAT

TGGTCCAGCCACCAGGGGGGCCGACTTGGCCATTAAGTAATTTAGATATTTCCATAAATT

ATAATAGGTTTCTTAGGGGTCAATTTAGTC

**Primer:**

**F: ATGGCGAGCACTGCTCTGTC**

**R: GTAGTTCCACACCTTCTCAT**
