# Supplementary material for: Salt-Induced Damage is Alleviated by Short-Term Pre-Cold Treatment in Bermudagrass (Cynodon dactylon)
Source: Plants (Basel). 2019 Sep 13;8(9):347. doi: 10.3390/plants8090347 (PMC6784090; doi:10.3390/plants8090347)
Supplement: Supplementary file 1 [file plants-08-00347-s001.zip › supplementary material/table S1.docx]

**Table S1 Parameters of JIP test analysis in bermudagrass under different treatments.**

| Treatment  Parameters | CK | CA | salt | CA+salt |
| --- | --- | --- | --- | --- |
| Specific energy fluxes (per Q_A_ reducing PSII reaction centre -RC) | | | | |
| TR_0_/RC | 1.99±0.379a | 1.79±0.450b | 1.75±0.396b | 1.95±0.235a |
| ET_0_/RC | 0.95±0.203a | 0.91±0.251ab | 0.82±0.212c | 0.88±0.156bc |
| RE_0_/RC | 0.36±0.121b | 0.53±0.085a | 0.29±0.119c | 0.30±0.064bc |
| DI_0_/RC | 0.61±0.115b | 0.59±0.066bc | 0.79±0.082a | 0.56±0.075c |
| Quantum yields and efficiencies/probabilities | | | | |
| φP_0_ | 0.77±0.037a | 0.75±0.028b | 0.71±0.034c | 0.76±0.022ab |
| ψE0 | 0.48±0.053ab | 0.51±0.027a | 0.45±0.025c | 0.47±0.033b |
| φE0 | 0.37±0.034a | 0.38±0.031a | 0.32±0.023c | 0.35±0.022b |
| δR0 | 0.38±0.085b | 0.58±0.051a | 0.33±0.079c | 0.37±0.040bc |
| φR0 | 0.14±0.027b | 0.22±0.024a | 0.11±0.028c | 0.13±0.013bc |
| γRC | 0.28±0.036b | 0.30±0.040a | 0.27±0.037b | 0.30±0.029a |
| RC/ABS | 0.39±0.073ab | 0.42±0.080a | 0.37±0.076b | 0.43±0.061a |
| Performance indexes (products of terms expressing partial potentials at steps of energy bifurcations) | | | | |
| PI_ABS_ | 1.16±0.220ab | 1.33±0.331a | 0.74±0.187b | 1.19±0.126ab |
| PI_total_ | 0.72±0.382b | 1.81±0.394a | 0.37±0.332c | 0.69±0.275b |

Mean values and standard deciation (SD) were calculated from three independent experiments. Different letters indicated significant difference (P ˂ 0.05) based on one-way analysis of variance (ANOVA), Duncan’s multiple range test. CK = control; NA = non-cold treatment; CA = pre-cold treatment for 6 h.
