# Supplementary figures and images for: Salt-Induced Damage is Alleviated by Short-Term Pre-Cold Treatment in Bermudagrass (Cynodon dactylon)
Source: Plants (Basel). 2019 Sep 13;8(9):347. doi: 10.3390/plants8090347 (PMC6784090; doi:10.3390/plants8090347)

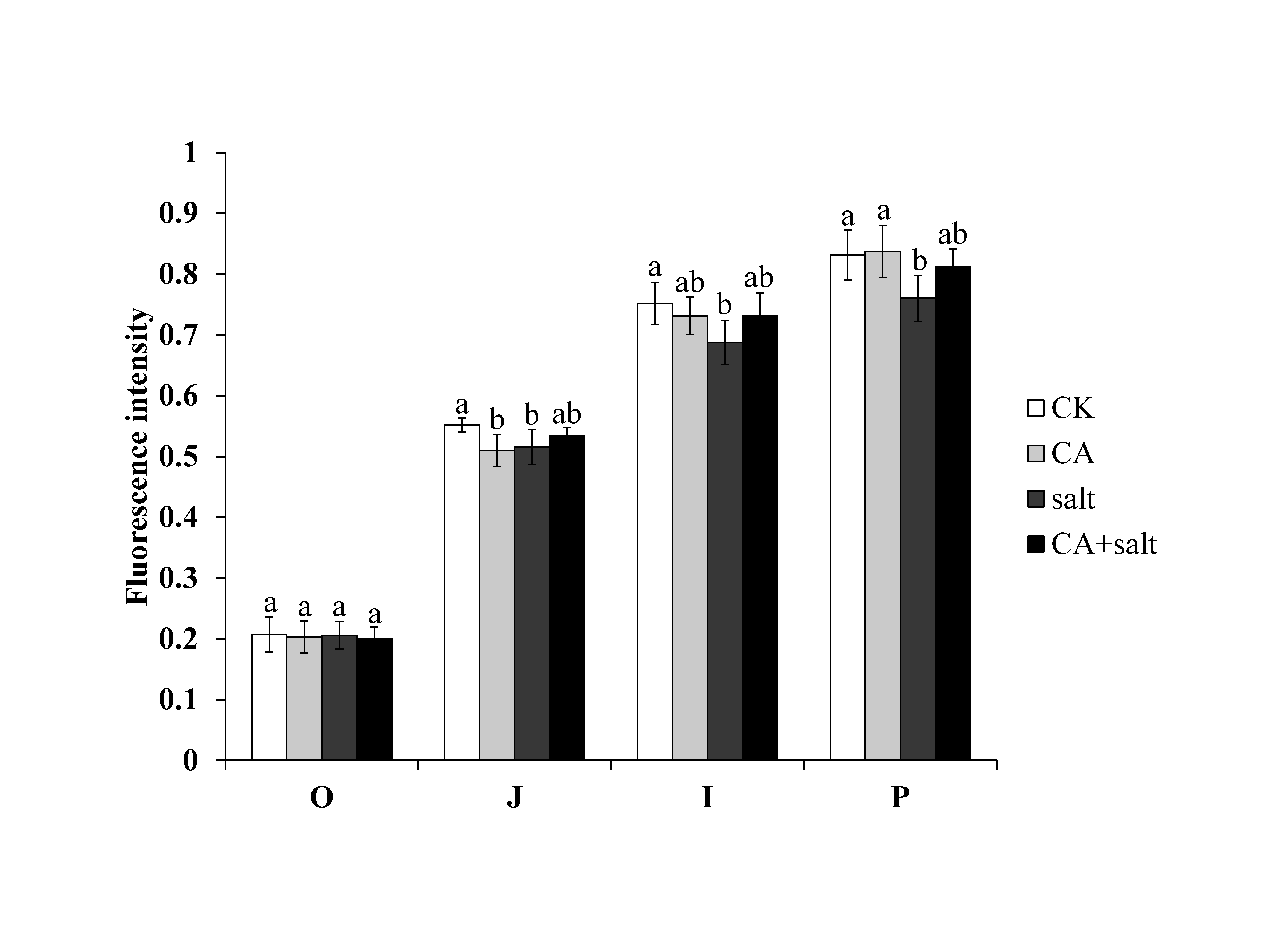

Supplement: Supplementary file 1 [file plants-08-00347-s001.zip › supplementary material/fig S1.tiff]
